# Sense of Coherence and Physical Health. Testing Antonovsky's Theory

**DOI:** 10.1100/tsw.2006.351

**Published:** 2006-10-09

**Authors:** Trine Flensborg-Madsen, Søren Ventegodt, Joav Merrick

**Affiliations:** ^1^ Quality of Life Research Center, Teglgårdstræde 4-8, DK-1452 Copenhagen K, Denmark; ^2^ Research Clinic for Holistic Medicine, Copenhagen, Denmark; ^3^ Nordic School of Holistic Medicine, Copenhagen, Denmark; ^4^ Scandinavian Foundation for Holistic Medicine, Sanvika, Norway; ^5^ Interuniversity College, Graz, Austria; ^6^ National Institute of Child Health and Human Development, Jerusalem, Israel; ^7^ Office of the Medical Director, Division for Mental Retardation, Ministry of Social Affairs, Jerusalem, Israel

**Keywords:** Antonovsky, sense of coherence, operationalization, physical health, comprehensibility, manageability, meaningfulness, Denmark

## Abstract

In a previous paper, we argued that the original 29-item sense of coherence (SOC) scale developed by Aaron Antonovsky (1923–1994) was insufficient according to its reflection of SOC. The purpose of this study was to create a new version of the original 29-item SOC scale in order to test his hypothesis of a causal link between SOC and physical health. This shorter version was built on the exact same idea, theory, and conceptualization used by Antonovsky, which resulted in a SOC scale containing only 9 abstract questions. These nine questions, in addition to two questions about physical and psychological health, made up a questionnaire answered by 100 people at the entrance hall of the University Medical Center (Rigshospitalet) in Copenhagen. According to Antonovsky’s famous hypothesis, a strong association should be found between SOC and physical health, but surprisingly, we found that the new scale was falsifying the hypothesis, with a correlation between SOC and physical health of only r = 0.044 (NS). However, a highly significant correlation was found with psychological health with r = 0.502 (*p* = <0.0005). The authors are in a predicament since we strongly believe in Antonovsky’s famous idea of the relationship between SOC and health. However, we believe that it is our emotional aspects that primarily determine our physical health, which we will demonstrate in a subsequent study, but the reason we did not find any significant correlation in this study was the fact that our nine-item SOC scale was very mental (mental in the sense of applying to conscious cognition and attitude). We consider the mental aspects to determine our psychological health and the emotional aspects to determine our physical health. Our conclusion is that the original 29-item SOC scale mixed a few emotional aspects into the otherwise mental construct, which is the reason for the relatively low correlations found until now, when using the original scale.

## INTRODUCTION

In 1970, Aaron Antonovsky (1923–1994), from the Faculty of Health Science at the Ben Gurion University of the Negev in Beer-Sheva, started to investigate what he would later name the salutogenic paradigm. His idea of salutogenesis was based on findings about the overall health of women who had been in a concentration camp during the Holocaust. Antonovsky advocated a salutogenic view as a contrast to the pathogenic view when discussing health and disease. In the salutogenic view, health is defined as a continuum between the two poles of total wellness and total disease, and Antonovsky suggested that the individual degree of sense of coherence in life influenced his or her position on this continuum:

> *"The central thesis of the salutogenic model is that a strong sense of coherence (SOC) is crucial to successful coping with the ubiquitous stressors of living and hence to health maintenance"[1].*

> *"My hypothesis, then, is that the strength of the SOC has direct physiological consequences and, though such pathways, affects health"[1].*

The idea of the salutogenic orientation proposed by Antonovsky has been well embraced by the established medical world, and several studies have shown, in fact, that his idea of salutogenesis is very reliable when represented though his 29- or 13-item SOC scale. However, we were surprised to find a lack of evidence when it came to providing an association between SOC and physical health[2], since most studies investigate the association with psychological health[3,4,5,6,7,8,9,10,11,12,13] or with health measures that incorporate psychological aspects[14,15,16,17].

In a previous paper, we analyzed Antonovsky's SOC scale (29- and 13-item SOC scale), and argued that some of the questions did not really express the key concepts of his thinking[18]. We suspect that it is the less than optimal operationalization of the theory into the 29-item SOC scale that caused the low or lacking correlations between SOC and physical health. In this study, we wanted to start once more, using Antonovsky's original theory to construct our own SOC scale, which had to be as clear, simple, and meaningful as possible.

## METHODS

Antonovsky was interested in factors that could describe the ability to manage the stressors that we are bombarded with every day, and he defined this as the "sense of coherence" (SOC):

> *"The sense of coherence is a global orientation that expresses the extent to which one has a pervasive, enduring though dynamic feeling of confidence that (1) the stimuli deriving from one's internal and external environments in the course of living are structured, predictable, and explicable [comprehensibility]; (2) the resources are available to one to meet the demands posed by these stimuli [manageability]; and (3) these demands are challenges, worthy of investment and engagement [meaningfulness]"*[1].

Hence, the "sense of coherence" is described in terms of the three dimensions: comprehensibility, manageability, and meaningfulness. Antonovsky stated that it is *not* necessary to feel that all of life is highly comprehensible, manageable, and meaningful in order to have a strong SOC. It is indeed possible to have a strong SOC and at the same time not see the entire world as coherent. This is due to the fact that all people set boundaries, and what goes beyond these simply does not matter to us, whether comprehensible, manageable, or meaningful or not. However, it is crucial that there are spheres of life that are of subjective importance to your life, and that these spheres are seen as comprehensible, manageable, and meaningful. Antonovsky defined four spheres from which it is not possible to narrow the boundaries; these are your inner feelings, immediate interpersonal relations, major activities, and existential issues. Basically, the idea of Antonovsky can be put in the following scheme (Table 1), where we have also generated the questions:

**TABLE 1**
**Basic Questions Constructed on the Basis of Antonovsky's Theory**

| | Comprehensibility | Manageability | Meaningfulness |
|---|---|---|---|
| **Inner feelings** | How well do you understand your feelings? | How well do you manage your feelings? | How meaningful do you experience your inner feelings? |
| **Immediate interpersonal relations** | How well do you understand your interpersonal relations? | How well do you manage your interpersonal relations? | How meaningful do you experience your interpersonal relations? |
| **Major activities** | How well do you understand the external world? | How well do you manage your external world? | How meaningful do you experience your external world? |
| **Existential issues** | How well do you understand your existence? | How well do you manage to handle your existence? | How meaningful do you experience your existence? |

The authors propose that the four spheres (from which Antonovsky suggested that it is not possible tonarrow the boundaries) can be limited to three spheres: self, life, and external world. This is based on theidea that "self" corresponds to "inner feelings", "life" corresponds to "existential issues", and "externalworld" corresponds to "major activities". The sphere "interpersonal relations" is not included as a separate sphere, since we believe it is covered by the other three; 'interpersonal relations is a functional perspective on something that is covered structurally by the other three spheres.The abbreviated matrix of questions is presented in Table 2.

**TABLE 2**
**United Questions Based on Table 1**

| | Comprehensibility | Manageability | Meaningfulness |
|---|---|---|---|
| **Self** | How good is your understanding of your self? | How well do you manage your self? | How meaningful do you experience your self? |
| **Life** | How good is your understanding of your life? | How well do you manage your life? | How meaningful do you experience life? |
| **External world** | How good is your understanding of your external world? | How well do you manage your external world? | How meaningful do you experience your external world? |

## Construction of the Questionnaire

A questionnaire was constructed that included the nine questions presented in Table 2 together with two questions concerning health; all questions were answered on a five-point Likert scale. Examples of some of the questions are shown in Table 3.

**TABLE 3**
**Examples of Three Questions in the Questionnaire**

| | | |
|---|---|---|
| **Question 1 and 2: As to your health, how do you feel now?** ***(Please circle a number in each column)*** | | |
| | Physical Health | Mental Health |
| Very well | 1 | 1 |
| Well | 2 | 2 |
| Neither well nor ill | 3 | 3 |
| Ill | 4 | 4 |
| Very ill | 5 | 5 |
| **Question 3: How good is your understanding of your self?** | | |
| Very good | 1 | |
| Good | 2 | |
| Neither good nor bad | 3 | |
| Bad | 4 | |
| Very bad | 5 | |

## The Sample

As a pilot experiment, the questionnaires were distributed to randomly selected persons at the entrance hall at the University Medical Center (Rigshospitalet) in Copenhagen, where the responders included patients, visitors, nurses, physicians, and other staff. Out of about 115 people, 100 people agreed to fill out the questionnaire. The questionnaires were filled out anonymously with time alone, and they were all put in a big envelope by the repliers. All the 100 questionnaires returned were filled out completely.

## Statistics

The SOC scale was constructed by adding the nine SOC questions into one measure from which it was possible to score between 9 and 45 points, with 9 implying a very high SOC and 45 implying a very low SOC. All nine items in the scale were positively correlated, and Cronbach's Alpha was found to be 0.755. The distribution of respondents according to SOC-9 is shown in Fig. 1, which illustrates that SOC-9 is approximately normal distributed around a mean score of 18.78 points and a standard deviation of 5.19.

Because of the relatively scarce degree of information, the relatively simple analyses Pearson correlation coefficient and Gamma correlation coefficient were used to describe the associations between SOC and physical and psychological health. All analyses were carried out using SPSS version 12.

## RESULTS

The mean SOC score was 18.78 points (standard deviation = 5.19), mean score for physical health was 2.35 (standard deviation = 1.10), and the mean score for psychological health was 2.10 (standard deviation = 1.01).

Table 4 illustrates that no significant correlation was found between SOC and physical health (r = 0.044; NS). However a strong correlation was found between the SOC and psychological health (r = 0.502; $p < 0.0001$).

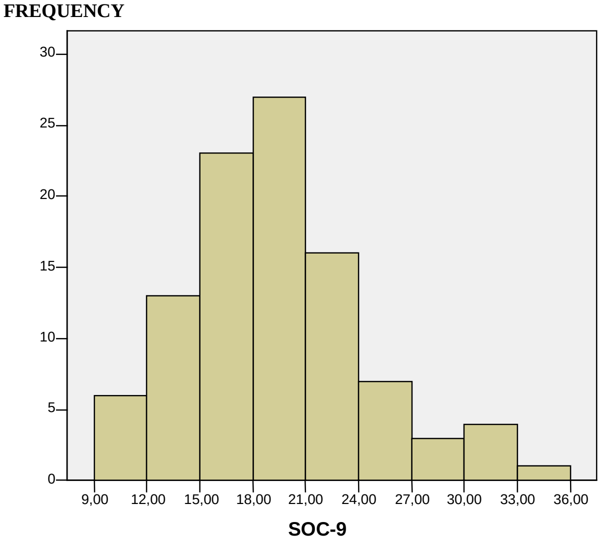

**FIGURE 1.** Distribution of respondents according to SOC.

**TABLE 4**
**Correlations Between the 9-Item SOC Scale and Health**

| | Physical Health | Psychological Health |
|---|---|---|
| Pearson correlation | 0.044 (NS) | 0.502 |
| Gamma correlation | 0.057 (NS) | 0.428 |

## DISCUSSION

As far as we know, this is the first study to test Antonovsky's original theory of SOC. Given results from various studies that have shown modest associations between the 29-item SOC scale and physical health[19,20,21,22,23,24,25,26], we expected that our new nine-item SOC scale would show the same tendencies. We also expected the association to be stronger, since our scale is based on the original idea, theory, and conceptualization as Antonovsky, but we have eliminated the notion of predictability, a factor that we concluded in a previous paper to be a disturbing factor in the 29-item SOC scale[18,27]. We were therefore surprised to see that there was no correlation whatsoever between SOC and physical health, since the correlation was only 0.044 (NS).

Since this is a pilot study with only 100 people and 11 questions in the questionnaire, we cannot be sure what the conclusion would be of a full-scale evaluation. It has not been possible to adjust for confounders, since we had no available information about gender, age, social group, or other demographic data. Moreover, the cross-sectional design was the main shortcoming of this study, thus leaving results open to interpretation.

It can be argued that something might be wrong with the formulation of the questions. However, one important statement can be made: The questions used in this short version of the SOC scale were also the basis for the 29-item SOC scale made by Antonovsky, so it cannot be the foundation of the questions that is wrong. Nevertheless, it can be argued that the formulation is very abstract and that the responders did not understand the questions in the same way, due to the fact that people have different levels of existential awareness. However, this does not seem to be the case, since we found a rather high correlation between SOC and psychological health of 0.502 ($p < 0.0001$). In addition, we found some very interesting results in a subsequent study that investigated the emotional sense of coherence (SOC-E)[28]. In that study, the questions were exactly as abstract as the ones that we used in the present study, however, our results were reverse since we found a significant correlation between SOC-E and physical health of –0.349 ($p < 0.01$) and an insignificant correlation between SOC-E and psychological health of only 0.188 (NS). Therefore, we believe that the majority of the responders understood the basic concepts and visions in every question in the way they were meant to understand them.

One interpretation of our work could be that we have actually falsified Antonovsky's hypothesis of a causal connection between *physical health* and the *sense of coherence* as expressed by the concepts of comprehensibility, manageability, and meaningfulness. However we found a high association between SOC and psychological health, so it is not health at large, but only physical health that gives us the difficulties. This might be the reason why most studies did not find associations between SOC and health, unless they focused on psychological health or did not distinguish clearly enough between physical and psychological health.

The original theory and conceptualization as presented by Antonovsky was very mental; meaning that it was intellectual and situated in the conscious mind. When Antonovsky operationalized his idea into the 29-item SOC scale, it seems that he mixed his mental idea with emotional aspects; examples of this are:

19: "Do you have very mixed-up feelings and ideas?"
21: "Does it happen that you have feelings inside you would rather not feel?"[1]

Our hypothesis has also been put forward by Korotkov[3] in a prospective study, which claimed that the SOC scale was confounded with emotionality, since the effect of SOC on physical health was removed once emotionality was taken out. That study concluded that SOC failed to predict physical symptoms prospectively.

We are strong believers in the concept of coherence and its association to health. However, we believe that physical disease is often connected to emotional issues and, therefore, it is the emotionality and *not* the mentality that determines physical health, as we will demonstrate in a subsequent study[28]. Conversely, the mentality obviously determines psychological health. When we built our questionnaire on the original theory of Antonovsky, our questions became very mental and ended up with a SOC scale that was purely mental and not emotional. This could very well be the reason why we found the large correlation between SOC and psychological health, and why we did not find any association between SOC and physical health. Our hypothesis also explains why the various studies investigating associations between the 29-item SOC scale and physical health have found modest associations at most[2], since the 29-item SOC scale mix a few emotional aspects into an otherwise mentally dominated list of items.

Before recommending a complete conceptual renewal of Antonovsky, his hypothesis should be tested again using yet a new questionnaire with each of the nine questions condensed into five or so more concrete, questions, as we right now must suspect that the level of abstraction could be some of the reasons for the low correlation that we found between SOC and physical health. Studies should also include more questions about the respondents in order to control for confounders such as gender, age, and social group.

## CONCLUSION

In an earlier article, we argued that the original 29-item SOC scale made by Aaron Antonovsky was insufficient[18]. The purpose of this study was to create a new version of the scale in order to test his hypothesis of a causal link between SOC and physical health. This shorter version was built on the exact same idea, theory, and conceptualization as that used by Antonovsky. Due to this, we expected to find a strong correlation between SOC and physical health. In a pilot study, we tested the new scale on 100 people and, surprisingly, found that there was no correlation whatsoever between the SOC and physical health. However, we found a substantial association between SOC and psychological health.

In spite of the various confounders that may have influenced this study, we still believe we have raised doubt about the hypothesis posed by Antonovsky; that a causal link exists between the original theory of SOC and physical health. We conclude from this study that the original 29-item SOC scale mixed mental and emotional aspects, which is the reason for the relatively low correlations found in relation to physical health. We believe that physical health is primarily determined by our emotionality and that this is the reason for our findings in this study, since our nine-item SOC scale is mental and does not, as we realize, incorporate emotional aspects as the original 29-item SOC scale did. In order to create a SOC scale that determines physical health, it will have to be merely emotional, since it is emotionality that really determines our physical health, external events aside. This has been demonstrated in another paper[28].
